# Spin–orbit coupling and Jahn–Teller effect in *T*_*d*_ symmetry: an *ab initio* study on the substitutional nickel defect in diamond

**DOI:** 10.1098/rsta.2022.0310

**Published:** 2024-01-22

**Authors:** Gergő Thiering, Adam Gali

**Affiliations:** ^1^ HUN-REN Wigner Research Centre for Physics, Institute for Solid State Physics and Optics, Institute for Solid State Physics and Optics, PO Box 49, H-1525, Budapest, Hungary; ^2^ Department of Atomic Physics, Institute of Physics, Budapest University of Technology and Economics, Müegyetem rakpart 3,1111 Budapest, Hungary

**Keywords:** diamond, Jahn–Teller effect, nickel, optically detected magnetic resonance, density functional theory

## Abstract

We analyse the spin–orbit and Jahn–Teller interactions in $T_{d}$ symmetry that are relevant for substitutional transition metal defects in semiconductors. We apply our theory to the substitutional nickel defect in diamond and compute the appropriate fine-level structure and magneto-optical parameters by means of hybrid density functional theory. Our calculations confirm the intepretations of previous experimental findings that the 2.56 and 2.51 eV optical centres are associated with this defect. Our analysis of the electronic structure unravels possible mechanisms behind the observed optical transitions and the optically detected magnetic resonance signal, too.

This article is part of the Theo Murphy meeting issue ‘Diamond for quantum applications’.

## 1. Introduction

Nickel is a typical contaminant in high-temperature high-pressure synthesized (HPHT) diamonds that paved the way to observe various nickel-related optical signals in diamond in the past decades [1,2]. However, various assignments of optical signals to defect structures were only tentative assumptions. Recent advances in the development of *ab initio* methods made it feasible to revisit the optical centres with high prediction power and identify the microscopic structure of the optical centres. As a recent example, we have identified the nickel-vacancy complex by means of advanced density functional theory (DFT) methods [3] as the origin of the 1.4 eV photo-luminescence (PL) centre [4–6] that was previously associated with the positively charged nickel interstitial [7–10]. Nickel-vacancy has $D_{3d}$ symmetry where group theory analysis combined with DFT calculations showed the electron–phonon coupling within Jahn–Teller (JT) effect formalism intertwine with spin–orbit coupling, and the full complexity of the system should be considered to obtain the key magneto-optical parameters for identification of nickel-related optical centres [3]. In this paper, we apply this approach to the substitutional nickel defect (${\mathrm{Ni}}_{\text{s}}$) in diamond that has $T_{d}$ symmetry. ${\mathrm{Ni}}_{\text{s}}$ introduces d orbitals to the electronic structure that presumably is also a subject of spin–orbit coupling, and partial occupation of degenerate d states could lead to the JT effect. As we shall show below, a very general group theory analysis of degenerate orbitals in $T_{d}$ symmetry is required for understanding this type of defect system.

The ${\mathrm{Ni}}_{\text{s}}$ configuration in diamond was observed in the electron paramagnetic resonance (EPR) spectra that is labelled as W8 [11–13]. After the discovery of W8 EPR centre [11], the milestone experiments in the identification of ${\mathrm{Ni}}_{\text{s}}$ were (i) the fingerprint of ${\;}^{61}\mathrm{Ni}$ isotope in the EPR spectrum with the hyperfine interaction between the electron spin and I=3/2 nuclear spin in isotopically ${\;}^{61}\mathrm{Ni}$ enriched HPHT diamond samples [12] and (ii) observation of S=3/2 electron spin with hyperfine signals of four identical ${\;}^{13}$C I=1/2 nuclear spins [13]. The W8 EPR centre exhibits an isotropic g=2.0310±0.0005 tensor. These facts imply that the defect has $T_{d}$ symmetry. Furthermore, S=3/2 spin state indicates that it is a substitutional defect in the negative charge state, i.e. ${\mathrm{Ni}}_{\text{s}}^{-}$. This model was later confirmed by additional measurements and *ab initio* calculations [14–19]. The ground state of ${\mathrm{Ni}}_{\text{s}}^{-}$ is well understood: ${\mathrm{Ni}}_{\text{s}}$ introduces a five times degenerate d orbital of which level splits due to the tetrahedral crystal field of diamond as a triple degenerate one ($t_{2}$) lying in the fundamental band gap, whereas the double degenerate d orbital (e) is resonant with the valence band. The in-gap $t_{2}$ level is occupied by three electrons in the negative charge state which establishes the paramagnetic S=3/2 state. This electronic configuration is the ${\;}^{4}A_{2}$ orbital singlet multiplet state in $T_{d}$ symmetry.

The correlation between the Ni-related optical centres and the W8 EPR centre were studied either indirectly such as common appearance in the appropriate optical and EPR spectra with similar estimated concentrations or directly via optically detected magnetic resonance (ODMR).

In the former method, the correlation between the 1.883 and 2.51 eV Ni-related absorption centres and the W8 EPR centre was investigated. It was concluded that the 2.51 eV absorption centre is likely linked to the W8 EPR centre rather than the 1.883 eV absorption centre [20]. Initial uniaxial stress measurements indicated that a triple degenerate $T_{2}$ state is involved in the 2.51 eV absorption spectrum [21] which was later revisited and concluded that ${\;}^{4}A_{2}↔{\;}^{4}T_{2}$ optical transition is involved in the absorption process [22], being consistent with the ground state of ${\mathrm{Ni}}_{\text{s}}^{-}$. Although, the ${\;}^{4}T_{2}$ state is not optically allowed from ${\;}^{4}A_{2}$ ground state, but it was assumed that spin–orbit coupling between the ${\;}^{4}T_{2}$ and ${\;}^{4}A_{2}$ makes this transition partially allowed. The shape of the first peak exhibits a doublet feature separated by 1.5 meV [22]. They interpreted this feature to the spin–orbit splitting of the ${\;}^{4}T_{2}$ state. Besides this feature a replica shows up at around 16.5 meV above the zero-phonon-line (ZPL) energy which is broadened compared with the shape of the ZPL emission [22]. The origin of the replica was not explained.

In the latter method, the 2.56 eV PL centre could be directly linked to the W8 EPR centre via ODMR measurements at cryogenic temperatures [23,24]. Two individual features were observed in the ODMR spectrum excited by 325 nm laser in a 35 GHz microwave cavity [24]: (i) an electron spin resonance with the isotropic g=2.0324(5) associated with the W8 EPR centre with producing a PL spectrum in the ODMR contrast, which agrees well with the 2.56 eV PL spectrum in terms of the ZPL energy and the features in its phonon sideband; (ii) the hyperfine splitting originating from the ${\;}^{14}N$ I=1 nuclear spin in the P1 EPR centre, i.e. the neutral substitutional nitrogen donor, $N_{\text{s}}^{0}$ with g≈2.00, which produces a very broad Gaussian-shape PL spectrum in the ODMR contrast. The ODMR contrast of the S=1/2 P1 centre was explained by a donor–acceptor pair (DAP) model, where the unknown acceptor A interacts with the $N_{\text{s}}$ donor as

1.1 $$N_{\text{s}}^{0}+A^{0}\to N_{\text{s}}^{+}+A^{-}+h\nu \text{, }$$

where hν photon is emitted, for which the energy depends on the distance between the $N_{\text{s}}$ and the acceptor A, so it results in a broad fluorescence spectrum for the ensemble of DAPs. If the unknown acceptor is boron (acceptor level is at 0.37 eV above the valence band maximum (VBM)) then the fluorescence spectrum can be well explained by taking the huge reconstruction energy of the $N_{\text{s}}$ upon ionization into account [24]. It was speculated that electron spin resonance of the acceptor in the ODMR spectrum was not observed because if the acceptor state is an effective mass like, then the degeneracy of the top of the VBM is causing its EPR signal to be strongly strain broadened and difficult to detect, as in all cubic semiconductors [24]. Regarding the Ni-related ODMR feature, it is more difficult to come up with a unique model that explains the observation of the ground state EPR of Ni, in the luminescence. Nazaré *et al.* speculated [24] that one possibility is spin-dependent hole transfer from a distant acceptor to ${\mathrm{Ni}}_{\text{s}}^{-}$, producing an excited luminescent state of ${\mathrm{Ni}}_{\text{s}}^{0}$. We note that this may explain that the observed isotropic g-factor in the ODMR spectrum (2.0324) slightly differs from the g-factor in the W8 EPR spectrum (2.0310) [24]. It is worth to note too that the 2.56 eV PL centre can be activated though a broad excitation at 200±20 nm range (approx. 6.2±0.6 eV) efficiently as performed by photo-luminescence excitation measurements [25]. This suggests that carriers trapping is indeed involved behind the ODMR process of ${\mathrm{Ni}}_{\text{s}}$ defect. We note that the reported negative ODMR contrast of the 2.56 eV PL in [23] was about $10^{-5}$ in their experimental conditions (excitation wavelength of 365 nm applied in 36.2 GHz microwave cavity), which showed up as a positive contrast without reporting its magnitude in [24] in their experimental conditions (excitation wavelength of 325 nm applied in 35 GHz microwave cavity). This fact also implies that the ODMR process is not intrinsic to the ${\mathrm{Ni}}_{\text{s}}$ defect alone. Photo-EPR measurements were carried out in nickel doped diamonds and found that the intensity of W8 EPR signal decreases by photo-excitation with approximately 2.5 eV threshold energy which can be reinstated by photo-excitation with approximately 3.0 eV [26].

## 2. Methodology

We characterize ${\mathrm{Ni}}_{\text{s}}$ in diamond by plane wave supercell calculations within spin-polarized DFT as implemented in the vasp 5.4.1 code [27]. We determine the electronic structure within the Born–Oppenheimer approximation where the ions are treated as classical particles. We relaxed the atomic positions until the forces acting on the ions fall below $10^{-2}\;\mathrm{eV}\;{\text{Å}}^{-1}$. We embed the defect in a 512-atom diamond supercell and we sample the Brillouin-zone at the Γ-point. We used the projector-augmentation-wave-method (PAW) [28,29] as implemented in vasp. We used the standard PAW projector for carbon and Ni\_pv PAW projector for nickel that includes the 3p atomic orbitals as valence. We applied a plane wave cut-off energy at 370 eV for the plane wave basis that was already proven to be convergent for SiV defect and many other defect system in diamond [3,30–35]. We calculate the excited states with the constrained-occupation DFT method or ΔSCF method [36]. We employed the Heyd–Scuseria–Ernzerhof (HSE06) hybrid functional [37,38], which reproduces the experimental band gap and the charge transition levels in diamond or other group-IV semiconductors within 0.1 eV accuracy [36,39]. For the charged supercell we applied the Freysoldt–Neugebauer–van de Walle correction to the total energy [40]. We used our home-built code to compute the electron–phonon spectrum of the JT models that we successfully applied to other systems, too [33,35].

## 3. Results

We combine group theory description of the ${\mathrm{Ni}}_{\text{s}}$ defect with DFT calculations. We start with the calculated electronic structure and charge transition levels of the defect. We identify the most common charge states under typical experimental conditions in this section. We continue with the detailed discussion of the ground state manifolds of the most relevant charged states of the defect and then we describe the optically accessible excited states. Finally, we discuss our findings in light of the observed 2.51 eV absorption centre and the 2.56 eV PL centre including the possible mechanisms behind the ODMR signature.

### (a) Electronic structure of ${\mathrm{Ni}}_{\text{s}}$

**Figure 1.**
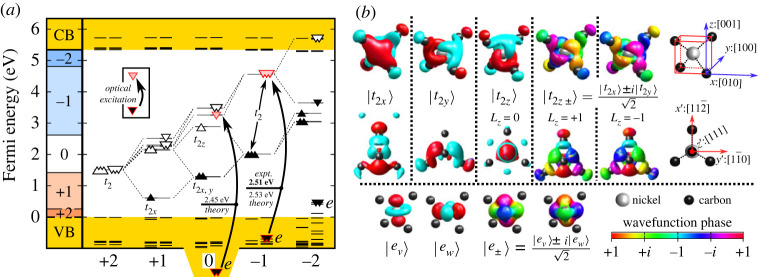
(*a*) Single particle levels and charge transition levels for ${\mathrm{Ni}}_{\text{s}}$. Note that the spin-polarization in the applied method splits the occupied (filled triangle, filled inverted triangle) and unoccupied (open triangle, open inverted triangle) defect levels, and the Fock-exchange potential in the hybrid DFT further lifts the degeneracy within the same spin channel for the occupied and unoccupied orbitals. Additionally, we show the lowest possible optical excitation between $t_{2}$ and e orbitals for ${\mathrm{Ni}}_{\text{s}}^{0}$ and ${\mathrm{Ni}}_{\text{s}}^{-}$. We note that the e orbitals ${\mathrm{Ni}}_{\text{s}}^{0}$ are smeared into multiple valence band states, thus its position is only approximate. However, upon ΔSCF procedure the e hole is getting localized and elevated into the band gap. (*b*) Kohn–Sham orbitals of ${\mathrm{Ni}}_{\text{s}}^{-}$ in diamond. In the real wave functions, the red and cyan isovalent surfaces correspond to positive and negative values, respectively. We plot the $|t_{2x,2y,2z}\rangle$ orbitals from ${\mathrm{Ni}}_{\text{s}}^{-}$’s triply degenerate (three filled triangles) occupied $t_{2}$ levels. The $|e_{u,w}\rangle$ are taken from ${\mathrm{Ni}}_{\text{s}}^{2-}$’s nearly doubly degenerate (two filled inverted triangles) occupied e orbitals.

The Ni atom introduces 3d orbitals which splits to double degenerate e orbitals and triple degenerate $t_{2}$ orbitals in the tetrahedral crystal field of diamond. In the defect molecule diagram, these 3d orbitals and the 4s orbitals interact with the four dangling bonds of the neighbour carbon atoms that results in $a_{1}$ and $t_{2}$ molecular orbitals under $T_{d}$ symmetry. The 4s and $a_{1}$ orbitals can form bonding and antibonding molecular orbitals lying deep in the valence band and high in the conduction band, respectively. The e orbitals do not recombine with the dangling bonds and they remain atomic like. The level of e orbitals is resonant with the valence band in the neutral charge state of the defect as plotted in figure 1*a*. Finally, the $t_{2}$ representations of the 3d orbitals and the dangling bonds again form bonding and antibonding molecular orbitals. The bonding combination falls deep in the valence band, whereas the antibonding combination appears in the gap. In the context, we call this antibonding $t_{2}$ orbital simply the $t_{2}$ state in the gap which is responsible for the ground state manifolds of the ${\mathrm{Ni}}_{\text{s}}$ defect. In the optical excitation, the atomic-like e states may play a role. The calculated Kohn–Sham states are depicted in figure 1*b*.

According to this defect molecule analysis, the defect has the $a_{1}^{2}t_{2}^{6}e^{4}t_{2}^{2-q}$ electronic configuration, where q is the charge state of the defect. As the triple degenerate $t_{2}$ may accommodate six electrons, the possible charge states may go from the (2+) to (4−). We calculated the total energies of the corresponding charge states ($E_{\text{tot}}^{q}$) and calculated the adiabatic charge transition levels as $(q|q-1)=E_{\text{tot}}^{q}-E_{\text{tot}}^{q-1}+qE_{\text{VBM}}$, where $E_{\text{VBM}}$ is the calculated VBM energy and now it is aligned to zero in figure 1*a*.

We find that the anticipated (3−) and (4−) states are not stable at all, and the (2+) and (2−) charge states are marginally stable as a function of the Fermi-level in diamond. Therefore, we do not analyse the (2+) and (2−) charge states further. The calculated (0|−) level is at about $E_{\text{VBM}}+2.6\;\mathrm{eV}$, which means that it is about $E_{\text{CBM}}-2.8\;\mathrm{eV}$, where $E_{\text{CBM}}$ is the energy of the conduction band minimum (CBM). In other words, if the defect was in the neutral charge state then about 2.6 eV photo-excitation energy is required to ionize to (−) charge state, and if the defect was in the (−) charge state then about 2.8 eV photo-excitation energy is required to re-ionize it back to the neutral charge state. ${\mathrm{Ni}}_{\text{s}}^{+}$ can be readily ionized to the neutral charge state by the photo-excitation threshold energy at about 1.5 eV. It can be read out from figure 1*a* that ${\mathrm{Ni}}_{\text{s}}^{+}$ may be stable in p-type diamond and near-infrared photo-excitation is needed below the photo-ionization threshold energy in order to observe its absorption or fluorescence signal. In practice, these conditions are not fulfilled, thus we will only briefly discuss the ground state of ${\mathrm{Ni}}_{\text{s}}^{+}$. We rather focus our discussion on the negatively charged and neutral ${\mathrm{Ni}}_{\text{s}}$ defects.

We briefly discuss now the 2.51 eV absorption and 2.56 eV PL centres in the light of the calculated photo-ionization threshold energies. According to the calculated (0|−) level at around $E_{\text{VBM}}+2.6\;\mathrm{eV}$, both optical centres might correspond to the neutral excitation of both ${\mathrm{Ni}}_{\text{s}}^{0}$ and ${\mathrm{Ni}}_{\text{s}}^{-}$ defects. The 2.56 eV PL centre has the ZPL energy just below the calculated ${\mathrm{Ni}}_{\text{s}}^{0}\to {\mathrm{Ni}}_{\text{s}}^{-}$ ionization threshold energy. Thus, the calculated ionization threshold energies could not distinguish alone which optical centre belongs to which defect charge state. In order to identify these optical centres, we analyse the ground and excited states of ${\mathrm{Ni}}_{\text{s}}^{-}$ and ${\mathrm{Ni}}_{\text{s}}^{0}$ in detail in the next sections.

It is intriguing that the 2.56 eV emission can be observed above-band-gap excitations [25], and its ODMR signals were observed by 325 nm (3.81 eV) photo-excitation [24] and 365 nm (3.40 eV) photo-excitation [23] experiments. In the latter two experiments, the photo-excitation energies are both above the ionization threshold energies for both the ${\mathrm{Ni}}_{\text{s}}^{0}\to {\mathrm{Ni}}_{\text{s}}^{-}$ and ${\mathrm{Ni}}_{\text{s}}^{-}\to {\mathrm{Ni}}_{\text{s}}^{0}$ processes, where the electron is promoted from the valence band to the empty defect level and from the occupied defect level to the conduction band, respectively. The calculated (0|−) level at around $E_{\text{VBM}}+2.6\;\mathrm{eV}$ implies that the electron is promoted from a deeper energy region of the valence band in the first process rather than it is excited from the occupied defect level to little above the CBM in the second process. As the electron density of states rapidly increases going further from the band edges this implies that the ${\mathrm{Ni}}_{\text{s}}^{0}\to {\mathrm{Ni}}_{\text{s}}^{-}$ process is much more efficient than the ${\mathrm{Ni}}_{\text{s}}^{-}\to {\mathrm{Ni}}_{\text{s}}^{0}$ process. As a consequence, the ultraviolet excitation will stabilize the ${\mathrm{Ni}}_{\text{s}}^{-}$ charge state, so the defect stays a much longer time in the (−) charge state than in the (0) charge state.

### (b) Ground state of ${\mathrm{Ni}}_{\text{s}}^{-}$

It is likely that ultraviolet photo-excitation stabilizes the negative charge state of ${\mathrm{Ni}}_{\text{s}}$, and the totally filled $t_{2}$ electronic configuration of ${\mathrm{Ni}}_{\text{s}}^{-}$ can be readily analysed as a basis for the more complex electronic configurations. Therefore, we start the analysis with the ground state electronic structure of ${\mathrm{Ni}}_{\text{s}}^{-}$.

According to the first Hund’s rule the three electrons in the triple degenerate $t_{2}$ orbitals will form a high-spin S=3/2 electronic configuration. The wave function of such quartet state can be expressed as

3.1 $${|}^{4}A_{2}\rangle =\underset{A|xyz\rangle }{\underbrace{A|t_{2x}t_{2y}t_{2z}\rangle }}⊗\left\{ \begin{array}{c} |↑↑↑\rangle \\ S|↑↑↓\rangle \\ S|↑↓↓\rangle \\ |↓↓↓\rangle \end{array}\right.\text{, }$$

where we define the three particle (S) symmetrization

3.2 $$S|↑↑↓\rangle =\frac{1}{\sqrt{3}}(|↑↑↓\rangle +|↑↓↑\rangle +|↓↑↑\rangle ),$$

and (A) anti-symmetrization

3.3 $$\begin{array}{rl} A|t_{2x}t_{2y}t_{2z}\rangle & \;=A|xyz\rangle \\ & \;=\frac{1}{\sqrt{6}}(|xyz\rangle -|xzy\rangle +|yzx\rangle -|yxz\rangle +|zyx\rangle -|zxy\rangle ), \end{array}$$

operators for the sake of simplicity. In addition, we use shortened (x,y,z) notation to label $\left(t_{2x},t_{2y},t_{2z}\right)$ orbitals in the context from now on. Our results are in agreement with previous *ab initio* studies [17,19], the ground state is a ${|}^{4}A_{2}\rangle$ multiplet. The S=3/2 spin of W8 EPR centre emerges from the three unpaired electrons filling the $t_{2}$ orbitals in ${\mathrm{Ni}}_{\text{s}}^{-}$.

### (c) Shelving doublet states of $t_{2}^{\left(3\right)}$ electronic configurations in ${\mathrm{Ni}}_{\text{s}}^{-}$

It is clear that $t_{2}^{\left(3\right)}$ electronic configuration can form doublet states too as the total number of possible combinations of multiplet states is (63)=20, where ${\;}^{4}A_{2}$ quartet state produces only four multiplet states from them. The other configurations form doublet manifolds that should be expressed as a combination of Slater-determinants. First, we determine the possible configurations with single Slater-determinants. There are eight possible occupations where we pin electrons on individual x,y,z spin–orbitals as

3.4 $$\begin{array}{rl} & A|x_{↑}y_{↑}z_{↑}\rangle ,\;A|x_{↑}y_{↑}z_{↓}\rangle ,\;A|x_{↑}y_{↓}z_{↑}\rangle ,\;A|x_{↓}y_{↑}z_{↑}\rangle \\ \mathrm{and}\;\; & A|x_{↓}y_{↓}z_{↓}\rangle ,\;A|x_{↓}y_{↓}z_{↑}\rangle ,\;A|x_{↓}y_{↑}z_{↓}\rangle ,\;A|x_{↑}y_{↓}z_{↓}\rangle \end{array}\},$$

where $S_{z}=\pm (3/2)$ combinations and the totally symmetric $S_{z}=\pm (1/2)$ combinations are reserved for the ${|}^{4}A_{2}\rangle$ ground state as

3.5 $${|}^{4}A_{2}\rangle =\left\{\begin{array}{c} A|x_{↑}y_{↑}z_{↑}\rangle \\ \frac{1}{\sqrt{3}}(A|x_{↑}y_{↑}z_{↓}\rangle +A|x_{↑}y_{↓}z_{↑}\rangle +A|x_{↓}y_{↑}z_{↑}\rangle )\\ \frac{1}{\sqrt{3}}(A|x_{↓}y_{↓}z_{↑}\rangle +A|x_{↓}y_{↑}z_{↓}\rangle +A|x_{↑}y_{↓}z_{↓}\rangle )\\ A|x_{↓}y_{↓}z_{↓}\rangle \end{array}\right\},$$

that is equivalent to equation (3.1). Therefore, the remaining four-dimensional subspace spans a ${|}^{2}E\rangle$ state as

3.6 $${|}^{2}E\rangle =\left\{\begin{array}{c} \frac{1}{\sqrt{6}}(2A|x_{↑}y_{↑}z_{↓}\rangle -A|x_{↑}y_{↓}z_{↑}\rangle -A|x_{↓}y_{↑}z_{↑}\rangle )\\ \frac{1}{\sqrt{2}}(A|x_{↓}y_{↑}z_{↓}\rangle -A|x_{↓}y_{↓}z_{↑}\rangle )\\ \frac{1}{\sqrt{6}}(2A|x_{↓}y_{↓}z_{↑}\rangle -A|x_{↓}y_{↑}z_{↓}\rangle -A|x_{↑}y_{↓}z_{↓}\rangle )\\ \frac{1}{\sqrt{2}}(A|x_{↑}y_{↓}z_{↑}\rangle -A|x_{↑}y_{↑}z_{↓}\rangle ) \end{array}\right\}.$$

By constraint occupation of the $t_{2}$ Kohn–Sham orbitals, the total energy of the system can be calculated by spin-polarized HSE06 functional as given in equation (3.4). The exchange-correlation functional of HSE06 may not be able to capture the high correlation which can be described as combinations of Slater-determinants and the resulting solutions will be not the true eigenstate of the doublet spin state.

With this caveat, we carried out *ab initio* calculations for the single Slater-determinant configuration, e.g. $A|x_{↑}y_{↑}z_{↓}\rangle$ of the Kohn–Sham $t_{2}$ states which yields 0.35 eV with respect to the calculated total energy of ${\;}^{4}A_{2}$ state. This was achieved by restricting the symmetry to $T_{d}$ symmetry. By enabling the symmetry reduction for the $A|x_{↑}y_{↑}z_{↓}\rangle$ electronic configuration, the energy gain has become negligible at 1.4 meV. We conclude that there will be no JT effect for the doublet configurations in the first order. The $A|x_{↑}y_{↑}z_{↓}\rangle$ determinant is a 33% admixture of ${|}^{4}A_{2}\rangle$ and 66% of ${|}^{2}E\rangle$ multiplets, thus we expect the level of ${|}^{2}E\rangle$ lying above that of ${|}^{4}A_{2}\rangle$ by roughly 0.35 eV.

Other possible configurations are

3.7 $$\begin{array}{cccc} A|x_{↑}x_{↓}y_{↑}\rangle & A|x_{↑}x_{↓}y_{↓}\rangle & A|x_{↑}x_{↓}z_{↑}\rangle & A|x_{↑}x_{↓}z_{↓}\rangle \\ A|y_{↑}y_{↓}z_{↑}\rangle & A|y_{↑}y_{↓}z_{↓}\rangle & A|y_{↑}y_{↓}x_{↑}\rangle & A|y_{↑}y_{↓}x_{↓}\rangle \\ A|z_{↑}z_{↓}x_{↑}\rangle & A|z_{↑}z_{↓}x_{↓}\rangle & A|z_{↑}z_{↓}y_{↑}\rangle & A|z_{↑}z_{↓}y_{↓}\rangle \end{array}\},$$

which reduces into a ${|}^{2}T_{1}\rangle$ spin doublet as

3.8 $${|}^{2}T_{1}\rangle =\left\{\begin{array}{c} A|x_{\sigma }y_{↓}y_{↑}\rangle -A|x_{\sigma }z_{↓}z_{↑}\rangle \\ A|y_{\sigma }z_{↓}z_{↑}\rangle -A|y_{\sigma }x_{↓}x_{↑}\rangle \\ A|z_{\sigma }x_{↓}x_{↑}\rangle -A|z_{\sigma }y_{↓}y_{↑}\rangle \end{array}\right\}⊗\left\{ \begin{array}{c} \sigma =↑\\ \sigma =↓ \end{array}\right.,$$

and another ${|}^{2}T_{2}\rangle$ spin doublet as

3.9 $${|}^{2}T_{2}\rangle =\left\{\begin{array}{c} A|x_{\sigma }y_{↓}y_{↑}\rangle +A|x_{\sigma }z_{↓}z_{↑}\rangle \\ A|y_{\sigma }z_{↓}z_{↑}\rangle +A|y_{\sigma }x_{↓}x_{↑}\rangle \\ A|z_{\sigma }x_{↓}x_{↑}\rangle +A|z_{\sigma }y_{↓}y_{↑}\rangle \end{array}\right\}⊗\left\{ \begin{array}{c} \sigma =↑\\ \sigma =↓ \end{array}\right..$$

We expect that the levels of these states lie above those of the maximally open shell configurations’ doublets because the Coulomb repulsion is not compensated by exchange interaction in the former. In the Kohn–Sham DFT method, we could calculate one of the single Slater-determinants electronic configuration as expressed in equation (3.7). Their total energy exceeds that of ${|}^{2}E\rangle$ by about 0.5 eV within $T_{d}$ symmetry.

Finally, we conclude based on the Coulombic repulsion principle that ${|}^{2}T_{2}\rangle$ has the highest total energy among the considered doublet manifolds as it exhibits the most symmetric polynomial, that is, $x(y^{2}+z^{2})$, in contrast to ${|}^{2}T_{1}\rangle$ with $x(y^{2}-z^{2})$.

### (d) $t_{2}^{\left(2\right)}$ electronic configurations in ${\mathrm{Ni}}_{\text{s}}^{0}$

The ground state manifolds of ${\mathrm{Ni}}_{\text{s}}^{0}$ exhibit $t_{2}^{\left(2\right)}$ electronic configurations in $T_{d}$ symmetry, which may be a subject of JT effect and spin–orbit coupling. The total number of manifolds is (62)=15. According to direct product tables [41] of $T_{d}$ point group, there are four multiplets that can be read as

3.10 $$t_{2}^{\left(2\right)}={\;}^{1}A_{1}⊕{\;}^{1}E⊕{\;}^{3}T_{1}⊕{\;}^{1}T_{2},$$

where we kept the fermionic anti-symmetric property of the wave functions, thus ${\;}^{3}A_{1}$, ${\;}^{3}E$, ${\;}^{1}T_{1}$, ${\;}^{3}T_{2}$ combinations do not occur. Next, we show the symmetrically adapted wave functions. According to our *ab initio* results the ground state of ${\mathrm{Ni}}_{s}^{0}$ is a ${|}^{3}T_{1}\rangle$ that reads as

3.11 $${|}^{3}T_{1}\rangle =\left\{\begin{array}{c} A|yz\rangle \\ A|zx\rangle \\ A|xy\rangle \end{array}\right\}⊗\left\{ \begin{array}{c} |↑↑\rangle \\ S|↑↓\rangle \\ |↓↓\rangle \end{array}\right.\text{.}$$

It is followed by the ${|}^{1}T_{1}\rangle$ singlet state, which can be expressed as

3.12 $${|}^{1}T_{2}\rangle =\left\{\begin{array}{c} S|yz\rangle \\ S|zx\rangle \\ S|xy\rangle \end{array}\right\}⊗A|↑↓\rangle ,$$

where we interchanged the two-particle $A|ab\rangle =\left(1/\sqrt{2}\right)(|ab\rangle -|ba\rangle )$ anti-symmetrization and $S|ab\rangle =\left(1/\sqrt{2}\right)(|ab\rangle +|ba\rangle )$ symmetrization operators on the spin and orbital part of the wave functions.

The triplet configuration can be determined by Kohn–Sham DFT as a $|x^{↑}y^{↑}\rangle$ configuration. We note that ${|}^{3}T_{1}\rangle$ triplet is JT unstable. We obtained 159 meV relaxation energy when we removed the $T_{d}$ symmetry constraint during the geometry optimization. We obtained 47 meV higher total energy for the $|x^{↑}y^{↓}\rangle$ with $T_{d}$ symmetry restriction that gained 125 meV relaxation energy upon removal of the symmetry constraint. We note that the $|x^{↑}y^{↓}\rangle$ configuration is the 50–50% admixture of the ${|}^{3}T_{1}\rangle$ and ${|}^{1}T_{2}\rangle$ multiplets, thus our results can be interpreted as that the singlets are above the triplet by about approximately 47 meV.

Equations (3.11) and (3.12) span a 9+3=12 dimensional open shell subspace where the electrons are placed to different orbitals within $t_{2}$ orbitals. Next, we discuss the case of the closed shell configurations (xx, yy, zz) which leads to the ${|}^{1}A_{1}\rangle$ and ${|}^{1}E\rangle$ multiplets which may read as

3.13 $${|}^{1}E\rangle =\left\{\begin{array}{c} \frac{1}{\sqrt{6}}(2|zz\rangle -|xx\rangle -|yy\rangle )\\ \frac{1}{\sqrt{2}}(|xx\rangle -|yy\rangle ) \end{array}\right\}⊗A|↑↓\rangle$$

and

3.14 $${|}^{1}A_{1}\rangle =\frac{1}{\sqrt{3}}(|zz\rangle +|xx\rangle +|yy\rangle )⊗A|↑↓\rangle ,$$

respectively. We note that ${|}^{1}E\rangle$ and ${|}^{1}A_{1}\rangle$ are multi-configurational states, thus Kohn–Sham DFT may not provide good estimates for their total energies. Nevertheless, we can determine the total energy within spin-averaged Kohn–Sham DFT calculation for the individual (xx, yy, zz) configurations that forces the same orbitals in each spin channel. According to our results, the total energy of the closed shell configurations lies above that of the ground state by 0.70 eV within $T_{d}$ symmetry. After removing the symmetry constraint in the geometry optimization procedure, the calculated energy difference is reduced to 0.18 eV that shows a giant 0.52 eV JT energy. This effect might suppress the electronic correlation energy between ${|}^{1}E\rangle$ and ${|}^{1}A_{1}\rangle$, similarly to the product JT effect in ${\mathrm{SiV}}^{0}$ defect of diamond [42,43]. In the next section, we discuss the JT effect in detail for $t_{2}^{\left(2\right)}$ electronic configuration in $T_{d}$ symmetry.

**Figure 2.**
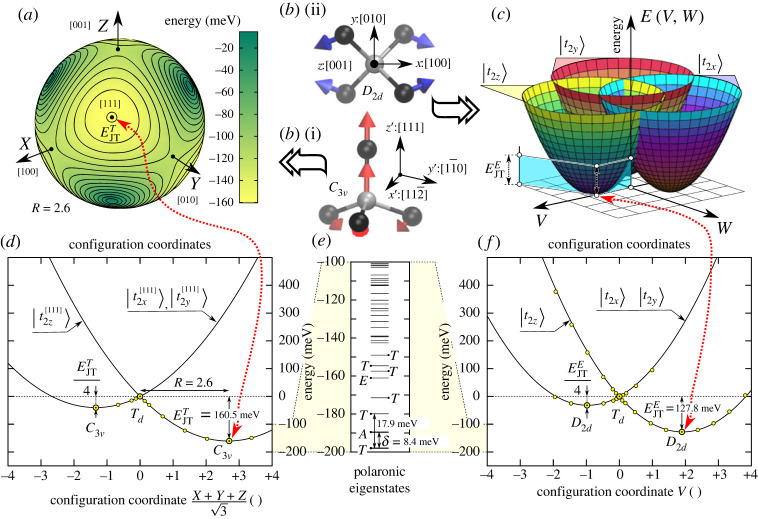
(*a*) Lowest layer of APES for T vibration mode showing the four equivalent minima. (*b*) Geometry distortions for (i) E and (ii) T type modes towards configuration coordinates V and $\left(X+Y+Z)/\sqrt{(}3\right)$, respectively. (*c*) APES for E vibration mode depicting the three equivalent minima points towards directions +V and $-(1/2)V\pm (\sqrt{3}/2)W$. (*d*) APES for T vibration mode towards the [111] distortion. Quantization axis and shape for $|t_{2\cdots }^{\left[111\right]}\rangle$ orbitals are shown in the second row of figure 1*b*. (*e*) Polaronic eigenspectrum by means of equation (3.15) for ${\mathrm{Ni}}_{\text{s}}^{0}$ without the $(3/2)\hbar \omega_{T}+(2/2)\hbar \omega_{E}$ zero point energy terms. (*f*) APES for E vibration mode towards the V distortion. Quantization axis and shape for $|t_{2\cdots }\rangle$ orbitals are shown in the first row of figure 1*b*. We note that yellow filled-in data points depict *ab initio* results.

#### (i) T⊗(t⊕e) Jahn–Teller effect in ${\mathrm{Ni}}_{\text{s}}^{0}$

The theory of JT interaction in $T_{d}$ symmetry was already discussed in the literature (see [44] and references therein). We apply this theory to the ${\mathrm{Ni}}_{\text{s}}^{0}$ defect. The $T_{d}$ symmetry is dynamically distorted by t and e vibration modes, i.e. T⊗(t⊕e) vibronic system. With assuming an effective t vibration and an effective e vibration, the vibronic system may read as

3.15 $$\begin{array}{rl} {\hat{H}}_{\mathrm{JT}} & \;={\hat{H}}_{\mathrm{JT}}^{T}+{\hat{H}}_{\mathrm{JT}}^{E}\\ & \;=\hbar \omega_{T}\sum_{n=X,Y,Z}\left(a_{n}^{†}a_{n}+\frac{1}{2}\right)+\hbar \omega_{E}\sum_{n=V,W}\left(a_{n}^{†}a_{n}+\frac{1}{2}\right)\\ & \;\;+\left[\begin{array}{ccc} F_{E}\left(\frac{1}{2}\hat{V}-\frac{\sqrt{3}}{2}\hat{W}\right) & -F_{T}\hat{Z} & -F_{T}\hat{Y}\\ -F_{T}\hat{Z} & F_{E}\left(\frac{1}{2}\hat{V}+\frac{\sqrt{3}}{2}\hat{W}\right) & -F_{T}\hat{X}\\ -F_{T}\hat{Y} & -F_{T}\hat{X} & -F_{E}\hat{V} \end{array}\right]\text{, } \end{array}$$

where $\hat{X},\hat{Y},\hat{Z}$ operators depict the geometry distortion from the t vibration mode governed by ${\hat{H}}_{\mathrm{JT}}^{T}$ while $\hat{V},\hat{W}$ depict to that of e vibration mode governed by ${\hat{H}}_{\mathrm{JT}}^{E}$. $F_{E}$ and $F_{T}$ electron-vibration coupling parameters can be determined [44] as

3.16 $$F_{T}=\sqrt{\frac{3}{2}E_{\mathrm{JT}}^{T}\hbar \omega_{T}}\;\text{and}\;F_{E}=\sqrt{2E_{\mathrm{JT}}^{E}\hbar \omega_{E}},$$

where the $E_{\mathrm{JT}}^{T,E}$ is the JT energy and $\hbar \omega_{T,E}$ is the respective effective phonon t and e frequencies. The global minima in the fivefold configurational space are either one of the three equivalent tetragonal extrema with $D_{2d}$ symmetry or four equivalent trigonal extrema with $C_{3v}$ symmetry. The actual global energy minima, whether tetragonal or trigonal, depend on the properties of the given system. As the t and e vibrations are orthogonal to each other and they lead to trigonal and tetragonal distortions, their individual contributions can be determined by DFT calculations as ,

3.17a $$E_{\mathrm{JT}}^{T}=E_{\mathrm{tot}}^{\mathrm{DFT}}(C_{3v})-E_{\mathrm{tot}}^{\mathrm{DFT}}(T_{d})$$

and

3.17b $$E_{\mathrm{JT}}^{E}=E_{\mathrm{tot}}^{\mathrm{DFT}}(D_{2d})-E_{\mathrm{tot}}^{\mathrm{DFT}}(T_{d}),$$

where $E_{\mathrm{tot}}^{\mathrm{DFT}}(\Gamma )$ total energies are obtained from HSE06 DFT calculations with geometry optimization under the Γ point group. We determined $\hbar \omega_{T,E}$ by mapping the parabolicity of the adiabatic potential energy surface along the e and t normal coordinates.

We solved the JT problem up to eight-phonon limit by substituting our *ab initio* parameters $E_{\mathrm{JT}}^{T}=160.5\;\mathrm{meV}$, $\hbar \omega_{T}=45.0\;\mathrm{meV}$, $E_{\mathrm{JT}}^{E}=127.8\;\mathrm{meV}$, $\hbar \omega_{E}=69.5\;\mathrm{meV}$ into equations (3.16) and (3.15). In accordance to previous results [44], the lowermost eigenvalue of the electron-vibration or vibronic system is a triple degenerate T level, which is followed by an A level by 9.0 meV (figure 2). ${|}^{3}T_{1}\rangle$ also experience spin–orbit effect as the maximum degeneracy in the $T_{d}$ double group is fourfold. In the next section, we study the spin–orbit interaction of this state.

#### (ii) Spin–orbit fine structure of ${\mathrm{Ni}}_{\text{s}}^{0}$

We introduce the spin–orbit operator as follows. The ${\hat{L}}_{x,y,z}$ operators depict an effective L=1 orbital moment within the $t_{2}$ orbitals. One may note that there are two electrons in the $t_{2}$ orbital, thus

3.18 $${\hat{H}}_{\mathrm{LS}}=\lambda_{0}\hat{L}\hat{S}=\lambda \left[\begin{array}{ccc} & -i{\hat{S}}_{z} & -i{\hat{S}}_{y}\\ i{\hat{S}}_{z} & & -i{\hat{S}}_{x}\\ i{\hat{S}}_{y} & i{\hat{S}}_{x} \end{array}\right],$$

where

3.19 $${\hat{L}}_{x}=\left(\begin{array}{ccc} 0\\ & 0 & -i\\ & i & 0 \end{array}\right)\;{\hat{L}}_{y}=\left(\begin{array}{ccc} 0 & & -i\\ & 0\\ i & & 0 \end{array}\right)\;{\hat{L}}_{z}=\left(\begin{array}{ccc} 0 & -i\\ i & 0\\ & & 0 \end{array}\right),$$

and ${\hat{S}}_{x,y,z}$’s are regular spin operators for a given spin multiplicity. For the triplet spin of ${\mathrm{Ni}}_{\text{s}}^{0}$, we use

3.20 $${\hat{S}}_{x}=\frac{1}{\sqrt{2}}\left(\begin{array}{ccc} & 1\\ 1 & & 1\\ & 1 \end{array}\right)\;{\hat{S}}_{y}=\frac{1}{\sqrt{2}}\left(\begin{array}{ccc} & -i\\ i & & -i\\ & i \end{array}\right)\;{\hat{S}}_{z}=\left(\begin{array}{ccc} 1\\ & 0\\ & & -1 \end{array}\right).$$

The complex i unit in ${\hat{L}}_{x,y,z}$ emphasizes that the $t_{2}$ orbitals lose their real-value character when spin–orbit interaction is considered, unlike the case of electron–phonon interaction, e.g. equation (3.15). For example, if the quantization axis is along the ‘z’ [001] axis then the eigenfunctions are $|ml=\pm 1\rangle =|t_{2x}\rangle \pm i|t_{2y}\rangle /\sqrt{2}$ and $|ml=0\rangle =|t_{2z}\rangle$ that we visualize in figure 1*b*.

We determined the bare intrinsic spin–orbit strength at single particle level by *ab initio* calculations: $\lambda^{\left(1\right)}=\langle t_{2z+}^{↑}|\lambda^{\left(1\right)}\hat{l}\hat{s}|t_{2z+}^{↑}\rangle -\langle t_{2z-}^{↑}|\lambda^{\left(1\right)}\hat{l}\hat{s}|t_{2z-}^{↑}\rangle =6.78\;\mathrm{meV}$. The single particle $\hat{l}$ operator can be depicted by the same matrices that of equation (3.19) in contrast to a single electron’s spin $\hat{s}$ that can be depicted by two-times-two Pauli matrices: ${\hat{s}}_{x}=(1/2)\left(\begin{array}{cc} & 1\\ 1 \end{array}\right){\hat{s}}_{y}=(1/2)\left(\begin{array}{cc} & -i\\ i \end{array}\right){\hat{s}}_{z}=(1/2)\left(\begin{array}{cc} 1\\ & -1 \end{array}\right)$. We note that $\lambda^{\left(1\right)}$ acts only between single particle levels but ${\mathrm{Ni}}_{\text{s}}^{0}$ is effectively a two-electron system, see equation (3.11). Therefore, the spin–orbit strength for the two-particle system will be $\lambda^{\left(1\right)}\langle t_{2z+}^{↑}t_{2z}^{↑}|A^{†}\hat{l}\hat{s}A|t_{2z+}^{↑}t_{2z}^{↑}\rangle =\lambda_{0}\langle^{3}T_{2}^{\left(mj=+2\right)}|\hat{L}\hat{S}|{\;}^{3}T_{2}^{\left(mj=+2\right)}\rangle$. In order to solve the previous equation, we introduce an 1/2 Clebsch–Gordan coefficient for $\lambda_{0}=\lambda^{\left(1\right)}/2$ because only $t_{2z\pm }^{↑}$ possesses both angular and spin moments, so $t_{2z}^{↑}$ exhibiting ml=0 remains intact from spin–orbit coupling.

However, the intrinsic value of spin–orbit coupling is reduced by the $p_{T_{1}}$ Ham reduction factor. That is, the $\hat{L}$ orbital operators are mediated by strong JT renormalization thus are subject to the so-called Ham reduction factors (see section 5.6 about reduction factors in [44] for details). In other words, the triply degenerate $t_{2}$ phonons can entangle the three $|t_{2z\pm }\rangle$, $|t_{2z}\rangle$ orbital characters. That is the ground state can be depicted on a Born–Oppenheimer basis by ladder operators acting on vibration modes: $|{\tilde{t}}_{2z+}\rangle =c|t_{2z+}\rangle ⊗|0\rangle +d|t_{2z-}\rangle ⊗a_{X}^{†}|0\rangle +\cdots$, where we depict only the first terms in the series expansion. Therefore, the electron–phonon entangled $|{\tilde{t}}_{2z+}\rangle$ will exhibit a reduced spin–orbit strength as the ground state is not a pure $|t_{2z+}\rangle$ electronic character anymore: $|t_{2z-}\rangle$ mixes in. The intrinsic orbital angular momentum will be partially quenched by the $p_{T_{1}}$ factor: ${\hat{L}}_{\mathrm{eff}}=p_{T_{1}}{\left\langle \hat{L}\right\rangle }_{\text{phonons}}$. Thus the observable spin–orbit coupling will be partially quenched too: $\lambda_{\mathrm{eff}}=p_{T_{1}}\lambda_{0}$. We report the reduction factor as $p_{T_{1}}=0.0349$ by means of equation (3.15) when we capped the phonon quanta as n≤9. Finally, the $\lambda_{\mathrm{eff}}$ acting for ${|}^{4}T_{1}\rangle$’s L=1 orbital and S=1 spin moments will be

3.21 $${\hat{H}}_{\mathrm{eff}}=\underset{=\lambda_{\mathrm{eff}}}{\underbrace{\frac{\lambda^{\left(1\right)}}{2}p_{T_{1}}}}{\hat{L}}_{\mathrm{eff}}\hat{S},$$

where $\lambda_{\mathrm{eff}}=0.12\;\mathrm{meV}$. Our positive $\lambda_{\mathrm{eff}}$ is in agreement with Hund’s rules: the lowermost state in a J=0 ($A_{1}$) singlet followed by J=1 ($T_{1}$) at $\lambda_{\mathrm{eff}}$ energy and J=2 ($T_{2}⊕E$) at $3\lambda_{\mathrm{eff}}$, where we depicted double group representations [45] for the $T_{d}$ symmetry in (⋯) parentheses. However, higher order spin–orbit terms may dominate over the linear $\lambda_{\mathrm{eff}}$ similarly to that of ${\mathrm{Ni}}_{\text{s}}^{-}$’s excited state that we will describe in equation (3.28) in the next section.

### (e) Optically excited states of ${\mathrm{Ni}}_{\text{s}}^{-}$

We turn to the interpretation of the optical spectra of Ni-related centres. First, we consider the optically allowed excited states of ${\mathrm{Ni}}_{\text{s}}^{-}$. To this end, one has to go beyond $t_{2}^{\left(3\right)}$ multiplets because no other quartet state can be formed than the ${\;}^{4}A_{2}$ ground state. As can be seen in figure 1*a*, a resonant e state occurs in the valence band from which an electron may be promoted to the empty $t_{2}$ state in the spin minority channel. This results in $e^{\left(3\right)}t_{2}^{\left(4\right)}$ electron configuration, which is equivalent to $e^{\left(1\right)}t_{2}^{\left(2\right)}$ hole configuration. We assume that the $t_{2}^{\left(2\right)}$ prefers the ${|}^{3}T_{1}\rangle$ multiplet. With this constraint, the $e^{\left(1\right)}t_{2}^{\left(2\right)}$ configuration reads as

3.22 $${\;}^{3}T_{1}{⊗}^{2}E={\;}^{4}T_{1}⊕{\;}^{4}T_{2}⊕{\;}^{2}T_{1}⊕{\;}^{2}T_{2}\text{.}$$

According to Hund’s first rule, we expect that the high-spin quartet states are more stable than the low-spin doublet states. We focus now on discussion of the optically allowed quartet states. In the construction of the quartet states in equation (3.22) we may start with the doublet E state, which can be expressed by ($x^{2}-y^{2}$; $2z^{2}-x^{2}-y^{2}$) quadratic polynomials. These polynomials are represented by the $|v\rangle =d_{x^{2}-y^{2}}$ and $|w\rangle =d_{2z^{2}-x^{2}-y^{2}}$ Ni 3d atomic orbitals, as depicted in figure 1*b*. The lowest order polynomial for triple degenerate representation under $T_{d}$ point group is linear. As a consequence, the quartet states should transform as fourth order polynomials. By applying projection operators in group theory one finds that {$xy(x^{2}-y^{2})$ ; $zx(z^{2}-x^{2})$ ; $yz(y^{2}-z^{2})$} belongs to the $T_{2}$ irreducible representation, whereas {$yz(2x^{2}-y^{2}-z^{2})$ ; $zx(2y^{2}-z^{2}-x^{2})$ ; $xy(2z^{2}-x^{2}-y^{2})$} belongs to the $T_{1}$ representation. These states may be expressed by using |v⟩ and |w⟩ as

3.23 $${|}^{4}T_{2}\rangle =\begin{array}{l} A|yz\left(\frac{1}{2}v+\frac{\sqrt{3}}{2}w\right)\rangle \\ A|zx\left(\frac{1}{2}v-\frac{\sqrt{3}}{2}w\right)\rangle \\ A|xyv\rangle \end{array}\}⊗\left\{ \begin{array}{c} |↑↑↑\rangle \\ S|↑↑↓\rangle \\ S|↑↓↓\rangle \\ |↓↓↓\rangle \end{array}\right.$$

and

3.24 $${|}^{4}T_{1}\rangle =\begin{array}{l} A|yz\left(\frac{1}{2}w-\frac{\sqrt{3}}{2}v\right)\rangle \\ A|zx\left(\frac{1}{2}w+\frac{\sqrt{3}}{2}v\right)\rangle \\ A|xyw\rangle \end{array}\}⊗\left\{ \begin{array}{c} |↑↑↑\rangle \\ S|↑↑↓\rangle \\ S|↑↓↓\rangle \\ |↓↓↓\rangle \end{array}\right..$$

It may be recognized that ${\;}^{4}T_{1}$ states can be constructed by swapping |v⟩ and w⟩ in the multiplets of ${\;}^{4}T_{2}$ states. Another important and surprising observation is that both ${\;}^{4}T_{2}$ and ${\;}^{4}T_{1}$ states may be described as a single Slater-determinant (see the last row in equations (3.23) and (3.24)). As a consequence, the total energy of ${\;}^{4}T_{2}$ and ${\;}^{4}T_{1}$ states can be principally estimated from the KS DFT ΔSCF method once the respective |xyv⟩ and |xyw⟩ electronic configurations could be converged in this procedure. We obtained 2.61 and 2.95 eV excitation energies w.r.t. the ${\;}^{4}A_{2}$ ground state’s energy within $T_{d}$ symmetry.

The electronic configurations and their energy levels may then be expressed by omitting the spin degrees of freedom as follows:

3.25 $${\hat{W}}_{e}=(\Xi +\Lambda )|{\;}^{4}T_{1}\rangle \langle {\;}^{4}T_{1}|+\Xi |{\;}^{4}T_{2}\rangle \langle {\;}^{4}T_{2}|+0|{\;}^{4}A_{2}\rangle \langle {\;}^{4}A_{2}|\text{, }$$

where we define the ${\hat{P}}_{2}$ and ${\hat{P}}_{1}$ projectors as

3.26a $$\underset{{\hat{P}}_{2}}{\underbrace{|{\;}^{4}T_{2}\rangle \langle {\;}^{4}T_{2}|}}=\frac{1}{4}\left(\begin{array}{cccccc} 1 & -\sqrt{3}\\ -\sqrt{3} & 3\\ & & 1 & \sqrt{3}\\ & & \sqrt{3} & 3\\ & & & & 4 & 0\\ & & & & 0 & 0 \end{array}\right)\;\begin{array}{c} \leftarrow A|yzv\rangle \\ \leftarrow A|yzw\rangle \\ \leftarrow A|zxv\rangle \\ \leftarrow A|zxw\rangle \\ \leftarrow A|xyv\rangle \\ \leftarrow A|xyw\rangle \end{array}$$

and

3.26b $$\underset{{\hat{P}}_{1}}{\underbrace{|{\;}^{4}T_{1}\rangle \langle {\;}^{4}T_{1}|}}=\frac{1}{4}\left(\begin{array}{cccccc} 3 & \sqrt{3}\\ \sqrt{3} & 1\\ & & 3 & -\sqrt{3}\\ & & -\sqrt{3} & 1\\ & & & & 0 & 0\\ & & & & 0 & 4 \end{array}\right)\begin{array}{c} \leftarrow A|yzv\rangle \\ \leftarrow A|yzw\rangle \\ \leftarrow A|zxv\rangle \\ \leftarrow A|zxw\rangle \\ \leftarrow A|xyv\rangle \\ \leftarrow A|xyw\rangle \end{array}\text{.}$$

However, we learnt from the ${\;}^{3}T_{1}$ of ${\mathrm{Ni}}_{\text{s}}^{0}$ that is subject to the JT effect. Since ${\;}^{4}T_{2}$ and ${\;}^{4}T_{1}$ inherit this electron configuration one can suspect that the JT effect is not negligible in ${\;}^{4}T_{2}$ and ${\;}^{4}T_{1}$ states of ${\mathrm{Ni}}_{\text{s}}^{-}$. Indeed, approximately 84 meV energy is released once we remove the $T_{d}$ symmetry constraint in our *ab initio* calculations during the ΔSCF procedure. In the next section, we study the JT effect in the ${\;}^{4}T_{2}$ and ${\;}^{4}T_{1}$ states using ${\hat{P}}_{2}$ and ${\hat{P}}_{1}$ projectors in equations (3.26*a*) and (3.26*b*).

#### (i) $\left(T_{1}⊕T_{2})⊗(t⊕e\right)$ Jahn–Teller effect

We already analysed the JT effect for the T⊗(t⊕e) system in §3d(i). We rely on this analysis, which becomes more complex with taking both $T_{2}$ and $T_{1}$ states that might also interact with each other through electron–phonon coupling that we describe by JT effect. This may be described as
3.27
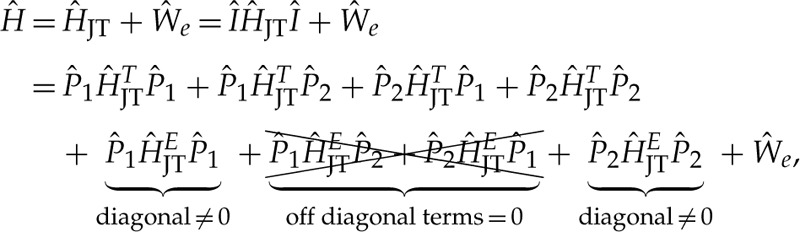
where we assume that ${\hat{H}}_{\mathrm{JT}}$ should not depend on |v⟩ and |w⟩ orbitals. That is, ${\hat{H}}_{\mathrm{JT}}$ in equation (3.27) is the dot product of ${\hat{H}}_{\mathrm{JT}}$ from equation (3.15) with a 2×2 unit matrix to span the six dimensional degrees of freedom from the orbitals. In the derivation, we used the lemma $\hat{I}=\hat{P_{1}}+\hat{P_{2}}$, which leads to diagonal and offdiagonal terms where we can distribute the JT interaction acting on inside the ${\;}^{4}T_{1,2}$ manifolds (*diagonal terms*) or induce orbital excitation between the ${\;}^{4}T_{1,2}$ multiplets. As a consequence, JT interaction induces mixing of electronic characters.

**Figure 3.**
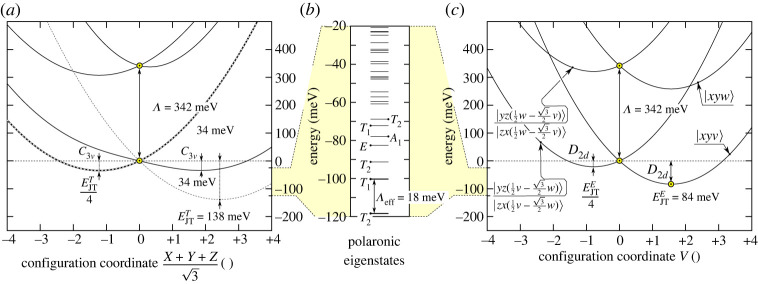
(*a*) APES for T vibration modes towards the [111] distortion for ${\mathrm{Ni}}_{\text{s}}^{-}$’s excited state. The dotted lines represent the APES where Λ=0 is assumed in equation (3.25). It can be seen that $E_{\text{JT}}^{T}/4$ minima at negative distortion is independent of the choice of Λ. Theoretically, it is possible to determine $E_{\text{JT}}^{T}$ directly by *ab initio* calculations; however, we were not able to obtain convergent results. Therefore, we assumed $E_{\text{JT}}^{T}=138\;\mathrm{meV}$ from ${\mathrm{Ni}}_{\text{s}}^{2-}$ charge state that exhibits the same electron occupancy in the $t_{2}$ orbital. We note that yellow filled-in data points are *ab initio* results. (*b*) Polaronic eigenspectrum by means of equation (3.27) for ${\mathrm{Ni}}_{\text{s}}^{-}$ without the $(3/2)\hbar \omega_{T}+(2/2)\hbar \omega_{E}$ zero point energy terms. (*c*) APES for E vibration modes towards the V distortion. Quantization axis and shape for $t_{2x,y,z}=x,y,z$ orbitals are shown in the first row of figure 1*b*.

First, one may check that the ‘E’ vibration modes do not lead to this kind of orbital mixing that we depicted in equation (3.27). That is, equations (3.26*a*) and (3.26*b*) show that the projectors are diagonal at their bottom-right edges, i.e. $\left(\begin{array}{cc} 0\\ & 4 \end{array}\right)$, $\left(\begin{array}{cc} 4\\ & 0 \end{array}\right)$, respectively. This bottom-right edge is influenced only by the $-F_{E}\hat{V}$ diagonal matrix element from equation (3.15) and thus does not inflict any interaction between A|xyv⟩ and A|xyw⟩ configurations of ${|}^{4}T_{2}\rangle$ and ${|}^{4}T_{1}\rangle$ multiplets, respectively. One may argue that the upper two 2×2 matrix blocks in equation (3.26) are not diagonal. However, these matrix blocks can be diagonalized with $A|xy((w/2)\pm (\sqrt{3}/2)v)\rangle$, $A|xy((v/2)\pm (\sqrt{3}/2)w)\rangle$ orbital rotations. Therefore, offdiagonal terms in equation (3.27) do not appear for E-type distortions. Therefore, APES remains the same for ${|}^{4}T_{1,2}\rangle$ states. They are just shifted by Ξ electronic splitting of equation (3.25) and thus equation (3.17*b*) can be used to determine $F_{E}$ strength. According to our *ab initio* calculations, the JT distortion energy is $E_{\mathrm{JT}}^{E}=84.2\;\mathrm{meV}$ mediated by $\hbar \omega_{E}=68.7\;\mathrm{meV}$ vibrations (figure 3*c*).

However, $F_{T}$ in equation (3.17*a*) is effective in this regard. $\hat{X}$, $\hat{Y}$, $\hat{Z}$ in equation (3.15) are purely offdiagonals that can create cross-talks between 2×2 blocks of the projectors in equation (3.26) and thus mix the ${|}^{4}T_{1,2}\rangle$ electronic characters since the three 2×2 blocks are not diagonal in the same basis. As we saw in the previous paragraph, the third block is diagonal in {v;w} basis but the first two blocks are diagonal only in $\left\{(v/2)\pm (\sqrt{3}/2)w,(w/2)\mp (\sqrt{3}/2)v\right\}$, thus ${\hat{H}}_{\mathrm{JT}}^{T}$ will cause orbital excitation between ${|}^{4}T_{1,2}\rangle$ multiplets. In other words, the APES will not be just a shifted replicas that of ${\mathrm{Ni}}_{s}^{0}$’s T⊗(t⊕e) JT APES depicted in §3d(i). Indeed, we report that the distortion energy is severely quenched for ‘T’-type distortions: $E_{\mathrm{JT}}^{T}=11.6\;\mathrm{meV}$ (that was 160 meV for ${\mathrm{Ni}}_{s}^{0}$) that demonstrates that equation (3.16) should not be directly employed for ${\mathrm{Ni}}_{s}^{-}$’s excited states. Therefore, we used $E_{\mathrm{JT}}^{T}=138.4\;\mathrm{meV}$ and $\hbar \omega_{T}=46.8\;\mathrm{meV}$ that of ${\mathrm{Ni}}_{s}^{2-}$ that resembles this excited state (figure 3*a*). Both configurations confine four electrons in $t_{2}$ orbitals thus we expect similar JT instability for these two cases approximately. We also assume that the pure atomic d orbitals in e states do not affect the JT instability too much.

Therefore, we take an exact diagonalization of equation (3.27) in order to approximate the vibronic levels up to n≤8 phonon limit. By considering the offdiagonal terms, the $|{\;}^{4}T_{2}\rangle$ and $|{\;}^{4}T_{1}\rangle$ can be entangled by phonons which will ultimately lead to a vibronically strongly coupled lowermost ${|}^{4}{\tilde{T}}_{2}\rangle$ polaronic state quickly followed by an additional ${|}^{4}{\tilde{T}}_{1}\rangle$ state at $\Lambda_{\mathrm{eff}}=18.0\;\mathrm{meV}$. We observe that at any choice for Λ below less than or equal to 342 meV the effective energy spacing will be strongly quenched as $\Lambda_{\mathrm{eff}}=p_{E}\Lambda$ by the $p_{E}$ Ham reduction factor. We note that $p_{E}$ affects orbital operators transforming as E representation of $T_{d}$ symmetry (see section 5.6 about reduction factors in [44] for details). In our present case, Λ energy exhibits the same order of magnitude that of $E_{\mathrm{JT}}^{T}$ or $E_{\mathrm{JT}}^{E}$ thus the two effects will compete with each other. As a consequence, $p_{E}$ will be dependent on Λ thus it is $p_{E}(\Lambda =342\;\text{meV})=18.02\;\text{meV}/342\;\text{meV}=0.0527$ in our case.

#### (ii) Spin–orbit fine structure

We learnt from ${\;}^{3}T_{1}$ ground state of ${\mathrm{Ni}}_{\text{s}}^{0}$ that spin–orbit interaction occurs. Nazaré *et al.* was able to fit the following spin–orbit Hamiltonian (see eqn (2) in [22]) to the fine structure of the 2.51 eV absorption centre,

3.28 $${\hat{H}}_{\mathrm{LS}}=\underset{=\lambda_{\mathrm{eff}}}{\underbrace{\lambda_{0}p_{T_{1}}}}{\hat{L}}_{\mathrm{eff}}\hat{S}+\kappa {\left({\hat{L}}_{\mathrm{eff}}\hat{S}\right)}^{2}+\rho \sum_{\alpha }^{x,y,z}{\hat{L}}_{\alpha }^{2}{\hat{S}}_{\alpha }^{2},$$

where the $\hat{L}$ orbital operators are that of equation (3.19) and $\hat{S}$ spin operators are extended for quartet (S=3/2) multiplicity to depict the spin–orbit interaction in a similar way to that of equation (3.18) for ${\mathrm{Ni}}_{\text{s}}^{0}$. However, in contrast to that of equations (3.18), (3.28) is an effective Hamiltonian in which the $\hat{L}$ orbital operators are mediated by strong JT renormalization, i.e. those are subject to the so-called Ham reduction factors. In this case, the reduction factor is $p_{T_{1}}$ because our three $\hat{L}$ orbital operators transform as the $T_{1}$ representation. Therefore, the intrinsic orbital angular momentum will be partially quenched by the $p_{T_{1}}$ factor: ${\hat{L}}_{\mathrm{eff}}=p_{T_{1}}{\left\langle \hat{L}\right\rangle }_{\text{phonons}}$ thus the observable spin–orbit coupling will be partially quenched too, i.e. $\lambda_{\mathrm{eff}}=p_{T_{1}}\lambda_{0}$. We report the reduction factor as $p_{T_{1}}=0.0604$ by means of equation (3.27) with using n≤7 for the expression of polaronic states.

The spin–orbit coupling parameter should be also determined. We were not able to converge the excited state together with spin–orbit coupling, thus we determined $\lambda_{0}$ by taking the value of ${\mathrm{Ni}}_{\text{s}}^{2-}$ that bears the same number of electrons on its $t_{2}$ orbital: $\lambda^{\left(1\right)}=\langle t_{2z+}^{↑}|\lambda^{\left(1\right)}\hat{l}\hat{s}|t_{2z+}^{↑}\rangle -\langle t_{2z-}^{↑}|\lambda^{\left(1\right)}\hat{l}\hat{s}|t_{2z-}^{↑}\rangle =-7.18\;\mathrm{meV}$. We note that $\lambda_{\left(1\right)}$, strictly speaking, acts only on single particle KS levels. Therefore, we introduce an additional 1/3 Clebsch–Gordan coefficient for $\lambda_{0}$: $\lambda^{\left(1\right)}\langle t_{2z+}^{↑}t_{2z}^{↑}e_{\pm }^{↑}|A^{†}\hat{l}\hat{s}A|t_{2z+}^{↑}t_{2z}^{↑}e_{\pm }^{↑}\rangle =(\lambda^{\left(1\right)}/3)\langle^{4}T_{2}^{\left(mj=+5/2\right)}|\hat{L}\hat{S}|{\;}^{4}T_{2}^{\left(mj=+5/2\right)}\rangle$. That is, in other words, neither $t_{2z}^{↑}$ nor $e_{\pm }^{↑}$ possesses $l_{z}$ angular momenta, thus only $t_{2z+}^{↑}$ couples with its ml=1 moment and ms=1/2 spin. In summary, we report $\lambda_{\mathrm{eff}}$ as $\lambda_{\mathrm{eff}}\approx (\lambda^{\left(1\right)}/3)p_{T_{1}}=-0.145\;\mathrm{meV}$ that is surprisingly near the experimentally observed −0.163 meV measured in the 2.51 eV absorption centre by Nazaré *et al.* [22].

However, our current level of theory was not able to determine the additional higher order κ and ρ parameters in equation (3.28). That is, determining those coefficients requires second order effects that would use ${|}^{2}T_{1}\rangle$, ${|}^{2}T_{2}\rangle$, ${|}^{2}E\rangle$ spin doublet states and even the ${|}^{4}A_{2}\rangle$ ground state through $∼\lambda_{\pm }^{\left(1\right)}|t_{2}^{↓}\rangle \langle e_{\;}^{↑}|$ flipping terms. We report $\lambda_{\pm }\approx 50\;\mathrm{meV}$ from *ab initio* calculations that would scale the ϱ,κ parameters by means of second order perturbation theory as

3.29 $$\varrho ,\kappa \propto \frac{{\left(\lambda_{\pm }/3\right)}^{2}}{E_{\text{tot.}}({\;}^{4}T_{2})-E_{\text{tot.}}({\;}^{2}T_{2})}\approx \frac{{(50/3)\;\text{meV}}^{2}}{1\;\text{eV}}=0.28\;\text{meV},$$

with assuming a heuristic approximately 1 eV electronic splitting. Our result in equation (3.29) is of the same order of magnitude that was reported in [22] as κ=−0.326 meV, ρ=+0.532 meV further supporting the association of 2.51 eV absorption centre to the ${\;}^{4}A_{2}\to {\;}^{4}T_{1}$ optical transition of ${\mathrm{Ni}}_{\text{s}}^{-}$.

### (f) Excited states of ${\mathrm{Ni}}_{\text{s}}^{0}$

We continue the investigation with the excited states of $e^{\left(1\right)}t_{2}^{\left(3\right)}$ electronic configuration which could be either $t_{2}^{↑↑↑}$ or $t_{2}^{↑↑↓}$ spin configurations coupled to ${\;}^{2}E$ which is a hole left on the d orbital with e symmetry with either ↑ or ↓ spin state. We start the analysis with the $t_{2}^{↑↑↑}$ case, which reads as

3.30 $${\;}^{4}A_{2}⊗{\;}^{2}E={\;}^{5}E⊕{\;}^{3}E\text{.}$$

The ${|}^{3}E\rangle$ is the case when the S=3/2 spin from ${|}^{4}A_{2}\rangle$ and the S=1/2 spin from the |e⟩ hole is coupled by the opposite spin states. The maximally spin-polarized wave functions are the following:

3.31 $$\begin{array}{rl} & \;{{|}^{5}E\rangle }_{ms=+2}=A|x_{↑}y_{↑}z_{↑}\xi_{↑}\rangle \\ & \;{{|}^{3}E\rangle }_{ms=+1}=\frac{1}{\sqrt{12}}(3A|x_{↑}y_{↑}z_{↑}\xi_{↓}\rangle -A|x_{↑}y_{↑}z_{↓}\xi_{↑}\rangle -A|x_{↑}y_{↓}z_{↑}\xi_{↑}\rangle -A|x_{↓}y_{↑}z_{↑}\xi_{↑}\rangle )\text{, } \end{array}$$

where the |ξ⟩ can be either |v⟩ or |w⟩. We note that the $A|x_{↑}y_{↑}z_{↑}\xi_{↓}\rangle$ single Slater-determinant dominates (75%) the ${{|}^{3}E\rangle }_{ms=+1}$ wave function, thus we expect that its total energy can be well estimated by the ΔSCF DFT method, which results in 2.61 eV excitation energy within $T_{d}$ symmetry. The JT energy is negligibly small (3 meV). The total energy of the ${\;}^{5}E$ state should be accurate by ΔSCF DFT method as a single Slater-determinant, and it yields 2.08 eV excitation energy. The JT energy is again tiny (5 meV) as expected.

We now turn to the other manifolds based on $t_{2}^{↑↑↓}$ electronic configurations, which result in ${\;}^{2}E$, ${\;}^{2}T_{1}$ and ${\;}^{2}T_{2}$ multiplets. We already proved during the discussion of ${\mathrm{Ni}}_{\text{s}}^{-}$ that ${\;}^{2}E$ multiplet exhibits the lowest total energy. Therefore, we only focus on that manifold as it is combined with a hole left on the d orbital with e symmetry which reads as follows:

3.32 $${\;}^{2}E⊗\;{\;}^{2}E={\;}^{3}A_{1}⊕{\;}^{3}A_{2}⊕{\;}^{3}E⊕{\;}^{1}A_{1}⊕{\;}^{1}A_{2}⊕{\;}^{1}E\text{.}$$

We assume again that the singlet manifolds lie at higher energies than the triplet manifolds. We express the maximal spin states of the optically allowed triplet manifolds,

3.33 $$\begin{array}{rl} & \;{|}^{3}A_{1}\rangle =A\left|w_{↑}\frac{1}{\sqrt{12}}(2x_{↑}y_{↑}z_{↓}-x_{↑}y_{↓}z_{↑}-x_{↓}y_{↑}z_{↑})+\frac{1}{2}v_{↑}(x_{↑}y_{↓}z_{↑}-x_{↓}y_{↑}z_{↑})\right\rangle \\ & \;{|}^{3}E\rangle =\left\{ \begin{array}{c} A\left|w_{↑}\frac{1}{\sqrt{12}}(2x_{↑}y_{↑}z_{↓}-x_{↑}y_{↓}z_{↑}-x_{↓}y_{↑}z_{↑})-\frac{1}{2}v_{↑}(x_{↑}y_{↓}z_{↑}-x_{↓}y_{↑}z_{↑})\right\rangle \\ A\left|w_{↑}\frac{1}{2}(x_{↑}y_{↓}z_{↑}-x_{↓}y_{↑}z_{↑})+\frac{1}{\sqrt{12}}v_{↑}(2x_{↑}y_{↑}z_{↓}-x_{↑}y_{↓}z_{↑}-x_{↓}y_{↑}z_{↑})\right\rangle \end{array}\right.\\ \mathrm{and}\; & \;{|}^{3}A_{2}\rangle =A\left|w_{↑}\frac{1}{2}(x_{↑}y_{↓}z_{↑}-x_{↓}y_{↑}z_{↑})-\frac{1}{\sqrt{12}}v_{↑}(2x_{↑}y_{↑}z_{↓}-x_{↑}y_{↓}z_{↑}-x_{↓}y_{↑}z_{↑})\right\rangle \end{array}\},$$

where the other spin states can be readily obtained and explicitly given here. It can be observed that these states are highly correlated. The largest determinant contribution is $(2/\sqrt{12}){\;}^{2}=33\%$ prefactors to where the ΔSCF DFT results can converge to

3.34 $${|}^{3}A_{1}\rangle ∼|w_{↑}x_{↑}y_{↑}z_{↓}\rangle \;\text{or}\;{|}^{3}A_{2}\rangle ∼|v_{↑}x_{↑}y_{↑}z_{↓}\rangle .$$

As a consequence, the total energies of these individual triplet states cannot be well determined by ΔSCF calculations. Nevertheless, we calculated the ΔSCF total energy of equation (3.34). We obtain 2.45 eV excitation energy within $T_{d}$ symmetry. The calculated JT energy is 43 meV, which is expected due to the partially filled $t_{2}$ orbitals. As the direct calculation of these states is not feasible with DFT methods, we do not analyse these states further but we give a very rough estimate for their ZPL energies at around 2.4 eV.

### (g) Ground state of ${\mathrm{Ni}}_{\text{s}}^{+}$

We briefly discuss paramagnetic ${\mathrm{Ni}}_{\text{s}}^{+}$, which may be observed in special p-type doped diamond sample. ${\mathrm{Ni}}_{\text{s}}^{+}$ exhibits $t_{2}^{\left(1\right)}$ electronic configuration, which yields $|{\;}^{2}T_{2}\rangle$ multiplet. This is subject to JT effect and spin–orbit interaction.

We can apply JT theory as given in equations (3.15) and (3.16), which results in $E_{\mathrm{JT}}^{T}=177.4\;\mathrm{meV}$, $\hbar \omega_{T}=46.8\;\mathrm{meV}$ and $E_{\mathrm{JT}}^{E}=115.2\;\mathrm{meV}$, $\hbar \omega_{E}=66.7\;\mathrm{meV}$. These results imply that the Ham reduction factor is significant that may act on the spin–orbit energy gaps. We determined $p_{T_{1}}=0.0209$ up to the 8-phonon limit. We determined the spin–orbit coupling $\lambda_{0}$ as 8.6 meV. We note here that no Clebsch–Gordan coefficient is needed in this case since the $|{\;}^{2}T_{2}\rangle$ multiplet can be described as a single $t_{2(\pm /0)}^{↑/↓}\rangle$ orbital. Therefore, the spin–orbit coupling will lead to an J=1/2 followed by an high moment J=3/2 state with $\lambda_{\text{eff}}=p_{T_{1}}\lambda_{0}=0.179\;\mathrm{meV}$. We again note that higher order spin–orbit terms may appear similarly to that of equation (3.28).

## 4. Discussion

**Figure 4.**
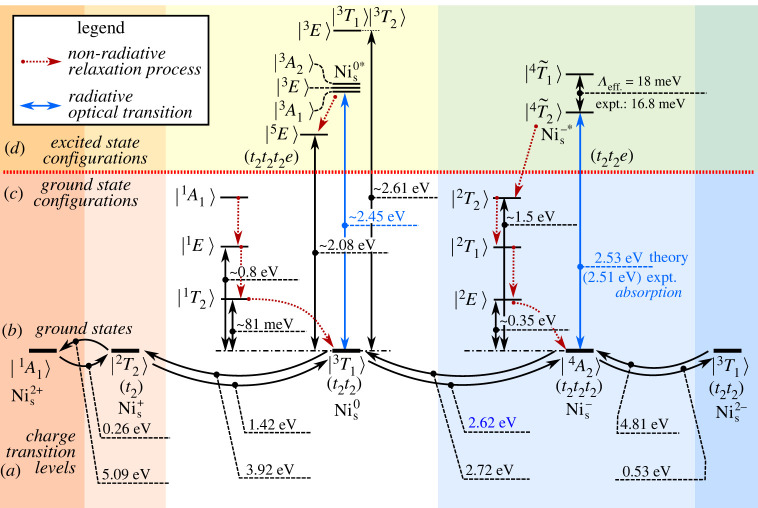
Ground (*b*) and excited states (*c* and *d*) of ${\mathrm{Ni}}_{\text{s}}$ defect in diamond in various charge states. The estimated ZPL energies of neutral excitation and photo-ionization threshold energies (*a*) are also given.

We summarize the calculated excitation energies and photo-ionization threshold energies of ${\mathrm{Ni}}_{\text{s}}$ defects in diamond in figure 4. As we can see, the neutral and negatively charged ${\mathrm{Ni}}_{\text{s}}$ has photo-ionization energies and excitation energies that are close to 2.5–2.7 eV, thus one has to study the nature of optical excitations in great detail. This includes the vibronic states and levels as well as the spin–orbit couplings and fine structure levels (figure 4). These results are the basis for the discussion of the origin of the 2.51 eV absorption centre and 2.56 eV ODMR centre. We note that the approximately 2.5 eV and approximately 3.0 eV threshold energies in the photo-EPR measurements [26] as the de-activation and re-activation of the W8 EPR centre associated with the ground state of ${\mathrm{Ni}}_{\text{s}}^{-}$ cannot be explained by simple ionization and re-ionization processes of ${\mathrm{Ni}}_{\text{s}}^{-}$, according to our results. In particular, we show below multiple evidences that the 2.51 eV absorption centre is associated with ${\mathrm{Ni}}_{\text{s}}^{-}$, thus the photo-ionization threshold energy towards the CBM cannot be smaller than 2.51 eV. It is rather likely that carriers trapping is also involved in the process where the carriers might be generated by photo-ionization of other defects in that diamond sample.

### (a) The 2.51 eV absorption centre and ${\mathrm{Ni}}_{\text{s}}^{-}$

We reiterate here that the 2.51 eV absorption centre is associated with a $|{\;}^{4}A_{2}\rangle \to |{\;}^{4}T_{2}\rangle$ optical transition according to the last observations which shows a replica at 16.5 meV above the ZPL feature [22]. Both the ZPL and the replica show a fine structure with about approximately 1.5 meV splitting where the ZPL’s fine structure is depicted by equation (3.28)’s effective spin–orbital Hamiltonian. The ${\mathrm{Ni}}_{\text{s}}^{0}$ has $T_{1}$ orbital ground state, whereas ${\mathrm{Ni}}_{\text{s}}^{-}$ has the appropriate $A_{2}$ ground state, thus we analyse the excited state of ${\mathrm{Ni}}_{\text{s}}^{-}$ as the possible candidate for the 2.51 eV absorption centre. Indeed, the calculated ZPL energy is at 2.53 eV, close to the experimental one. Additionally, we were able to predict the experimentally observed SOC parameter $\lambda_{\text{expt}}=-0.163\;\mathrm{meV}$ as $\lambda_{\text{eff}}=-0.145\;\mathrm{meV}$ suggesting that the model in [22] is correct.

We found that the lowest energy quartet excited state is indeed $|{\;}^{4}{\tilde{T}}_{2}\rangle$ for ${\mathrm{Ni}}_{\text{s}}^{-}$, which is optically forbidden in the first order, see figure 3*b*. The next quartet state is a ${\;}^{4}|{\tilde{T}}_{1}\rangle$ separated by about 18.02 meV from the $|{\;}^{4}{\tilde{T}}_{2}\rangle$ state that is very close to the observed replica at 16.5 meV. We emphasize that this effect is very similar that we found for the neutral silicon-vacancy centre in diamond [42] where two polaronically entangled states lie close to each other in the JT solution. An additional analogue to the properties of the neutral silicon-vacancy optical centre in diamond is that the first state is an optically forbidden ${\;}^{3}|{\tilde{A}}_{2u}\rangle$, which is quickly followed by the optically allowed ${\;}^{3}|{\tilde{E}}_{u}\rangle$ by approximately 7 meV that is an order of magnitude smaller than that of the vibration mode’s energy (ℏω=76 eV). A similar situation is apparent for ${\mathrm{Ni}}_{\text{s}}^{-}$: $\Lambda_{\text{expt}}=16.8\;\text{meV}<47\;\text{meV}=\hbar \omega_{T}$. Therefore, the vibration replica is significantly broader (approx. 5 meV, see fig. 1 in [22]) than that of the ZPL (approx. 2 meV) but not as broad as that of a real first phonon vibronic replica (approx. 30 meV) of Huang–Rhys theory [33,46]. Therefore, we argue that the 16.8 meV splitting is a fingerprint of the $\left(T_{1}⊕T_{2})⊗(t⊕e\right)$ JT instability.

The optical activity of the dark $|{\;}^{4}{\tilde{T}}_{2}\rangle$ state may be explained by the JT coupling to the bright $|{\;}^{4}T_{1}\rangle$ multiplet, which brings a $|{\;}^{4}T_{1}\rangle$ character to the $|{\;}^{4}{\tilde{T}}_{2}\rangle$ state of about 5% due to offdiagonal terms in equation (3.27). The remaining 95% of $|{\;}^{4}T_{1}\rangle$’s optical transition dipole moment would be still active around Ξ+Λ=2.51 eV+0.34 eV=2.85 eV (435 nm). However, this peak may be not recognized because it is simply obscured by the phonon sideband of the 2.51 eV peak and overlapping with the photo-ionization threshold at about 2.72 eV converting ${\mathrm{Ni}}_{\text{s}}^{-}$ to ${\mathrm{Ni}}_{\text{s}}^{0}$. Unfortunately, absorption data beyond greater than 2.54 eV (less than 488 nm) are not reported for the 2.51 eV centre, to the best of our knowledge, to gain insight about this issue.

Next, we discuss the strength of the ZPL and the replica. First, we note that even the ZPL of the lowermost $|{\;}^{4}{\tilde{T}}_{2}\rangle$ only contains 7% $|{\;}^{4}T_{1}\rangle$ contribution and 1% Debye–Waller factor, thus only 1% optical transition dipole moment is visible in the replica of 2.51 eV. Additionally, the $\lambda_{\pm }|t_{2}^{↑}\rangle \langle e^{↓}|$ spin–orbit coupling may turn $\langle^{4}A_{2}|\hat{d}|{\;}^{4}T_{2}\rangle$ optically allowed by ${\left(\lambda_{\pm }/\Xi \right)}^{2}\approx {\left(50\;\text{meV}/2510\;\text{meV}\right)}^{2}=0.04\%$ amount.

The missing fluorescence of the 2.51 eV band may be explained by the fact that the optical transition dipole moment is weak and ${|}^{2}T_{2}\rangle$, ${|}^{2}T_{1}\rangle$ and ${|}^{2}E\rangle$ doublets exist between the 2.51 eV optical transition that could lead to an efficient non-radiative channel for the ${\;}^{4}T_{2}$ excited state. The estimated spin–orbit coupling between the ${\;}^{2}E$ and ${\;}^{4}A_{2}$ states is about $\lambda_{\pm }∼50\;\mathrm{meV}$, which mediates the intersystem crossing in the non-radiative process.

### (b) The 2.56 eV ODMR centre and ${\mathrm{Ni}}_{\text{s}}^{0}$

The 2.56 eV ODMR centre is associated with the ground state of ${\mathrm{Ni}}_{\text{s}}^{-}$ but observed in luminescence which may be explained by the optical emission from ${\mathrm{Ni}}_{\text{s}}^{0}$ as speculated in [24]. We discuss this scenario in the light of our results. One of the ${\;}^{3}E$ excited states could be calculated with relatively high accuracy at 2.61 eV which is resonant with the calculated photo-ionization threshold energy at 2.62 eV. The excitation energy of the other three triplet excited states could not be well calculated by the ΔSCF method and it might fall below the photo-ionization threshold. However, the strength of optical transition associated with $|t_{2}\rangle \to |e\rangle$ at Kohn–Sham orbital level is very weak because the $t_{2}$ state is well localized on Ni 3d orbitals. We think that it is unlikely that those states are involved in the ODMR signal. We here sketch another scenario.

**Figure 5.**
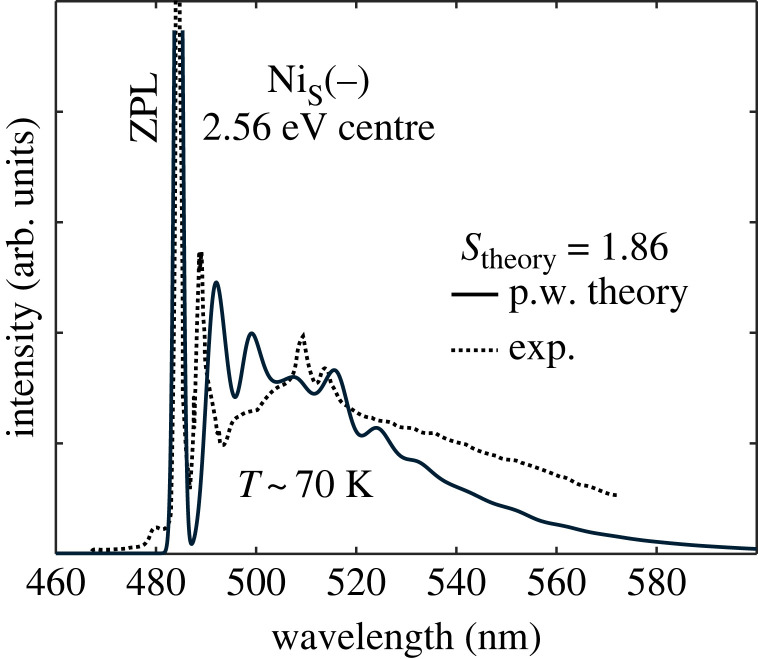
Huang–Rhys theory for the 2.56 eV PL/ODMR centre (exp.: [24]). Simulated ${\mathrm{Ni}}_{\text{s}}^{-}$+${\text{hole}}^{+}$ bound exciton → ${\mathrm{Ni}}_{\text{s}}^{0}$ fluorescence process. The calculated Huang–Rhys factor ($S_{\text{theory}}$) is also given.

As we discussed in §3a, ${\mathrm{Ni}}_{\text{s}}$ spends longer in the negative charge state upon ultraviolet illumination according to our calculations, and ultraviolet illumination also releases holes in diamond samples [24]. ${\mathrm{Ni}}_{\text{s}}^{-}$ can trap holes with very high hole capture rates that results in bound exciton states of ${\mathrm{Ni}}_{\text{s}}^{0}$. We note that ODMR signal via bound exciton excited states have been observed for the neutral silicon-vacancy defect [47], and it was argued for nitrogen-vacancy centre that bound exciton states have giant hole capture cross-section [48]. We analysed the bound exciton states for silicon-vacancy centre in $D_{3d}$ symmetry [47]. For ${\mathrm{Ni}}_{\text{s}}^{-}$ plus hole system, we should consider $T_{d}$ symmetry as the ${\mathrm{Ni}}_{\text{s}}^{-}$ ground state electronic configuration is stable in $T_{d}$ symmetry and the hole split from VBM should have negligible JT effect. For the hole state, we may use the effective mass theory as explained in [47]. The lowest energy state transforms by $A_{1}$ symmetry which has an 1s type envelope function. The 1s effective mass state is relatively well localized around the core of the defect for which the optical transition dipole moment is relatively strong. The phonon sideband of the fluorescence spectrum may be well estimated by the optimized geometries of the ${\mathrm{Ni}}_{\text{s}}^{-}$ representing the excited state and ${\mathrm{Ni}}_{\text{s}}^{0}$ for the ground state within Franck–Condon approximation. Indeed, the calculated and observed PL spectra agree well (figure 5). We conclude that the 2.56 eV PL and ODMR signals are associated with the bound exciton emission of ${\mathrm{Ni}}_{\text{s}}^{0}$.

Understanding the ODMR contrast requires the analysis of the fine structure of the ground state and the excited state. The ground state is ${\;}^{3}T_{1}$ which splits to J=0 ($A_{1}$) state, J=1 ($T_{1}$) state and J=2 ($T_{2}⊕E$) states in ascending energy order where we label here the double group representations. The splitting is caused by the spin–orbit interaction which is roughly estimated to be at 0.12 meV based on our *ab initio* calculations that do not contain quadratic spin–orbit contributions that might be also significant. Nevertheless, we assume that the order of these levels remains. In this case, the ground state has no effective coupling to the external magnetic field in the ground state, thus it may be not observable in EPR measurements. The JT nature of the ground state could also make it challenging to observe the triplet state in EPR experiments. In the excited state, the ${\;}^{4}A_{2}$ electronic configuration has $S_{1}=3/2$ spin state which is combined with a hole in the VBM with $S_{2}=1/2$ state ($T_{2}$). The composed system transforms at $T_{1}$ which again results in S=0 $A_{1}$ state, S=1 $T_{1}$ state and S=2 $T_{2}⊕E$ states split by spin–orbit interaction, where $S=S_{1}+S_{2}$ here. The initial population of the excited state branch may depend on the applied external magnetic field, temperature and strain in the sample. The final strength and sign of the ODMR contrast will depend on the population of these states in the excited state manifold. Further analysis of this process and the g-factor of the state is out of the scope of the present study.

### (c) Interaction of nickel defects with other defects in diamond

In the previous sections, we already outlined that defect–defect interactions play a crucial role in the manifestation of the ODMR signal associated with the substitutional nickel defect in diamond. Since the ionization energies of defects are much larger than the operation temperatures (typically, room temperature) the exchange of carriers is mediated by photo-excitation. The nitrogen donor can be efficiently activated by photo-excitation energy at 2.1 eV and above. The optical signals of substitutional nickel defects have ZPL energies at 2.51 eV and above, thus surrounding nitrogen donors around the nickel defects provide electrons towards the nickel defects upon illumination which may stabilize their negative charge state. On the other hand, if nickel-vacancy (NiV) defects are also present in the sample then this illumination results in ejecting holes by converting the 1.4 eV PL centre (negatively charged NiV defect) to doubly negative charged NiV defects (see [3]). Thus, the ratio and location of the nitrogen and NiV defects with respect to the substitutional nickel defects can be decisive about the magneto-optical fingerprints of the substitutional nickel defects. If vacancies or vacancy aggregates are also present in diamond, e.g. after implantation, then they also enter as traps for holes (in their negative charge states) and as sources of holes upon illumination of neutral vacancies or vacancy clusters. The latter can indeed occur as the calculated acceptor level of the vacancy or divacancy with respect to VBM is lower than 2.5 eV [49]. These defects and possibly boron acceptors are the major players in the photodynamics of the substitional nickel defect in diamond.

## 5. Summary

We studied the electronic structure of the Ni 3d transition metal substitutional defect with $T_{d}$ symmetry by means of group theory analysis and plane wave supercell DFT calculations. We find that the negative and neutral charge states are the most relevant configurations for the substitutional Ni defect in diamond. We observed strong electron–phonon coupling and spin–orbit coupling in certain states of the defect. We compared the *ab initio* results with the previously reported 2.51 eV absorption centre and the 2.56 eV ODMR centre in diamond, and we associate these centres to the negatively charged defect and emission from the bound exciton excited state of the neutral defect in diamond, respectively.

## Data Availability

Supporting datasets have been uploaded as electronic supplementary material: - data.xlsx contains the tables of data points plotted in the figures - VASP\_output.zip contains the key files of the *ab initio* simulations [50].
